# Clinicopathological analysis of composite lymphoma: A two-case report and literature review

**DOI:** 10.1515/med-2020-0191

**Published:** 2020-07-10

**Authors:** Wei Gui, Jing Wang, Li Ma, Yanli Wang, Liping Su

**Affiliations:** Department of Hematology, Shanxi Tumor Hospital, Taiyuan, Shanxi, People's Republic of China; Department of Pathology, Shanxi Tumor Hospital, Taiyuan, Shanxi 030013, People's Republic of China

## Abstract

**Objective:**

The objective of this study was to evaluate the clinicopathological features and treatment of composite lymphoma (CL) with cervical lymph node enlargement.

**Methods:**

In this study, two cases of CL are presented. Biopsies of enlarged cervical lymph nodes by excision revealed two distinct types of lymphomas. The diagnoses were confirmed by routine histopathology, immunohistochemistry, *in situ* hybridization, polymerase chain reactions and flow cytometry. Case 1 was diagnosed with Hodgkin’s lymphoma and cytotoxic T-cell lymphoma complicated by Epstein–Barr virus infection. Case 2 was diagnosed with diffuse large B-cell lymphoma and angioimmunoblastic T-cell lymphoma.

**Results:**

Case 1 received one cycle of adriamycin, bleomycin, vincristine and dacarbazine (ABVD regimen) combined with chidamide, followed by one cycle of gemcitabine and dexamethasone (GDP regimen) combined with chidamide, and then oral acyclovir. The patient achieved stable disease, but was lost to follow-up. Case 2 received eight cycles of cyclophosphamide, pirarubicin, vincristine and dexamethasone (CTOP regimen) combined with chidamide, and the patient achieved complete remission. Nine months later, relapse was confirmed. She received chidamide monotherapy for 3 months, which was then terminated. One year later, the patient underwent progressive disease and died.

**Conclusions:**

CL is a kind of rare disease. Due to the complexity of CL, clinicians should consider both disease components in order to increase the likelihood of effective treatment. This is important.

## 1 Case report

### 1.1 Case 1

**Figure 1:**
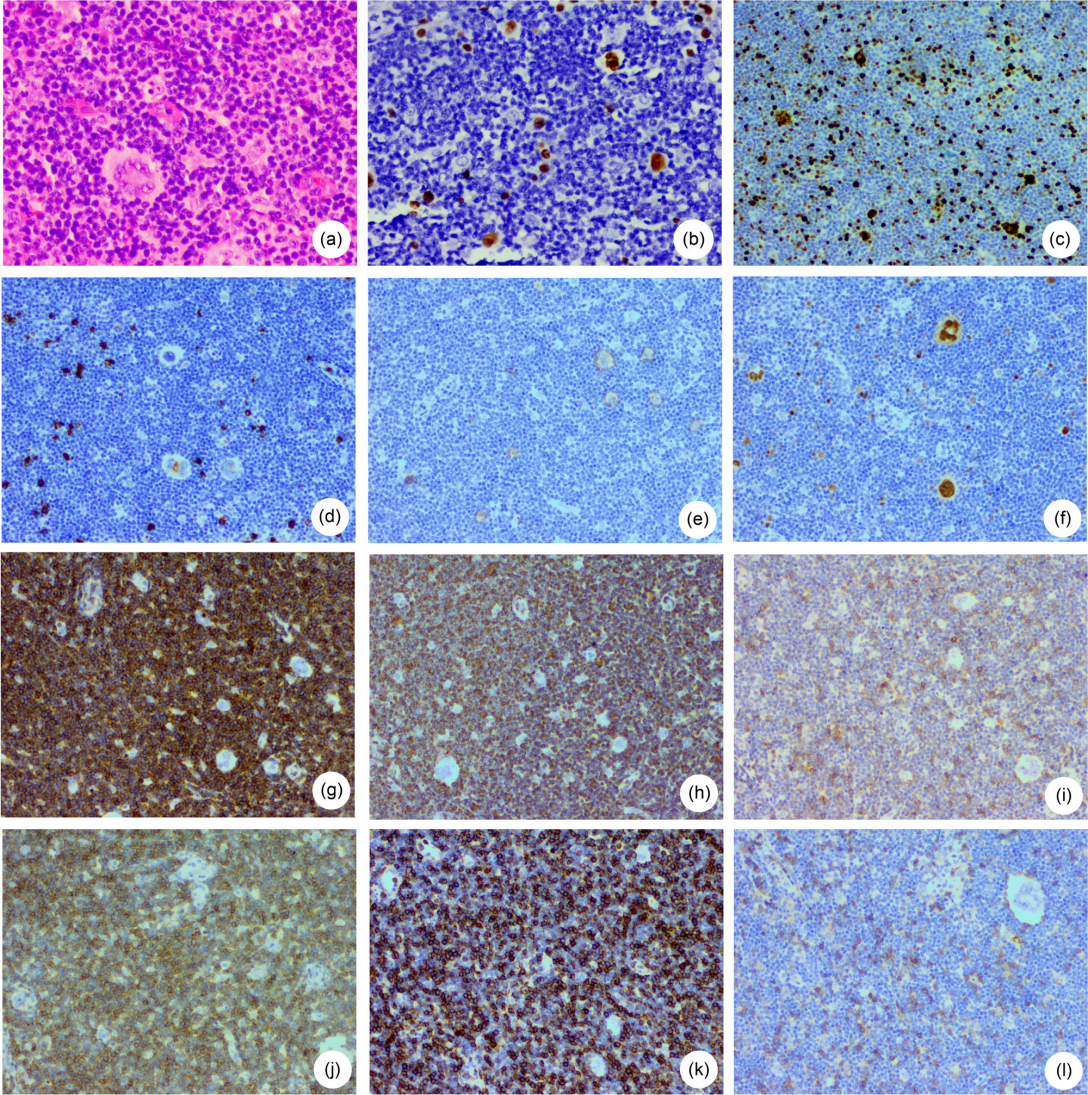
Histopathological findings of the lymph node biopsy performed on case 1 showing (a) R–S cells and scattered small T-lymphoid cells (hematoxylin and eosin, 200×), (b) EBER staining by *in situ* hybridization (100×), (c) R–S cells and T-lymphoid cells (40%) showing positive Ki-67 immunoreactivity (100×), (d) R–S cells showing weak CD15 immunoreactivity (100×), (e) R–S cells showing weak CD30 immunoreactivity (100×), (f) R–S cells showing positive MUM-1 immunoreactivity (100×), (g) T-lymphoid cells showing positive CD2 immunoreactivity (100×), (h) T-lymphoid cells showing positive CD3 immunoreactivity (100×), (i) T-lymphoid cells showing positive CD5 immunoreactivity (100×), (j) T-lymphoid cells showing weak CD7 immunoreactivity (40×), (k) T-lymphoid cells showing weak CD7 immunoreactivity (100×) and (l) T-lymphoid cells showing positive CD8 > CD4 immunoreactivity (40×).

**Figure 2:**
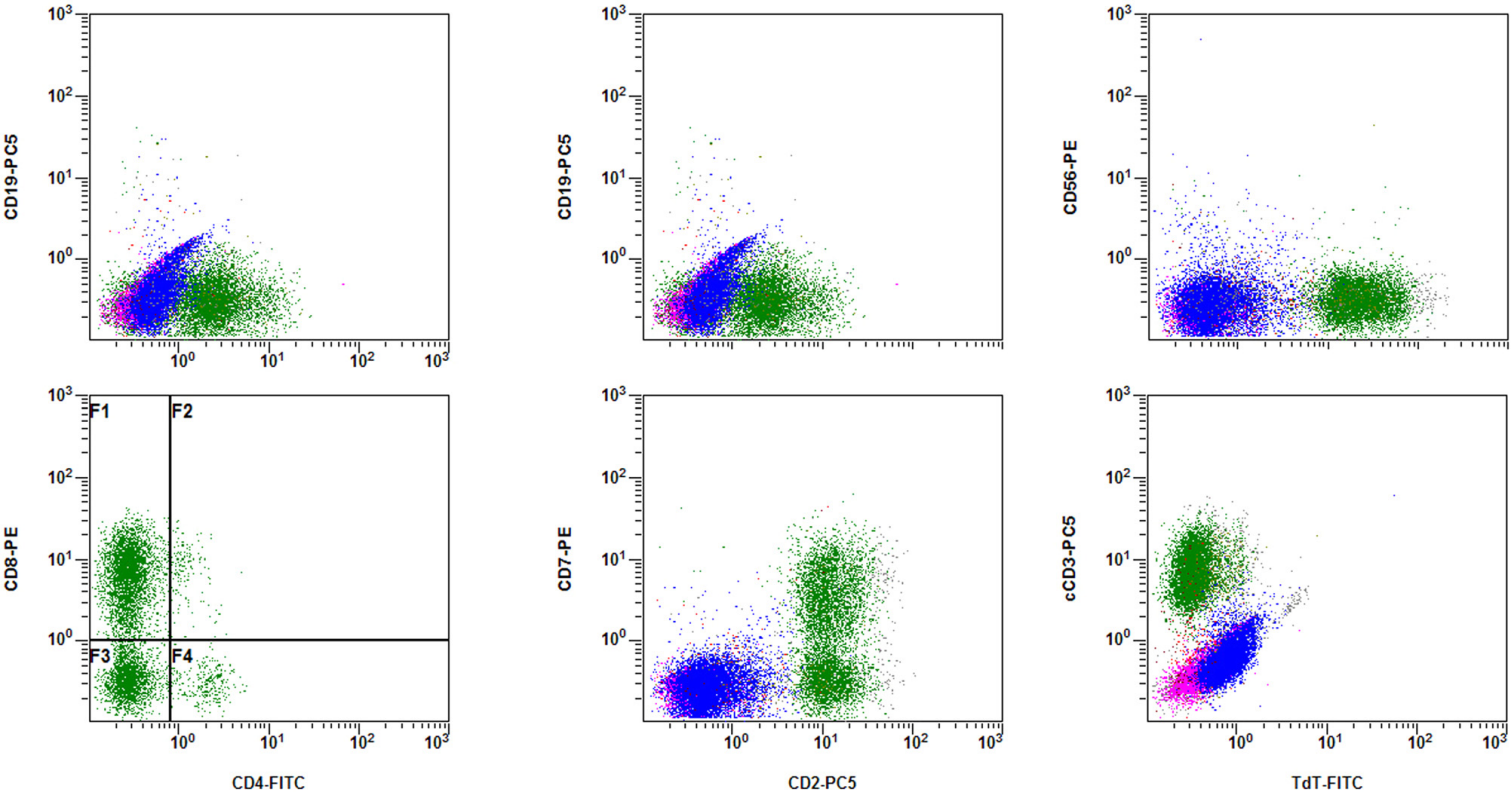
FCM results showing positive CD2, CD3, CD4, CD5, CD7 and CD8 immunoreactivity in BM cells of case 1.

A 44-year-old male was admitted to the Department of Head and Neck at Shanxi Tumor Hospital (Shanxi, China) in November 2017 with a 10-day history of a left cervical mass. Physical examination revealed an enlarged left cervical lymph node (4 cm × 1 cm). Laboratory findings showed a hemoglobin (HGB) level of 129 g/L, a white blood cell (WBC) count of 3.45 × 10^9^/L and a platelet (PLT) count of 118 × 10^9^/L. The levels of lactate dehydrogenase (LDH, 216 U/L) and beta2-microglobulin (β2-MG, 8.03 mg/L) were within normal ranges, and the results of liver and kidney functions and coagulation tests were normal. A positron emission tomography-computed tomography (PET-CT) scan revealed bilateral cervical, axillary, inguinal, mediastinal, and para-iliac vessels, as well as retroperitoneal lymphadenopathy and mild splenomegaly. A biopsy of the enlarged left cervical lymph node was performed. Histopathology showed diffuse infiltration of two different populations of lymphoid cells. Immunohistochemistry confirmed the co-existence of two malignancies, and polymerase chain reaction (PCR) results showed the expression of clonal T-cell receptor gamma (TCR-G) and beta (TCR-β). The first population of large lymphoid cells was characterized by vacuolar nuclei, eosinophilic nucleoli and a rich cytoplasm. The cells were immunopositive for CD15, CD30, PAX-5, MUM-1 and Ki-67 60%^+^, and they were characterized as Reed–Sternberg (R–S) cells. The second population of small lymphoid cells was characterized by heterotypic nuclei and condensed chromatin. The cells were immunopositive for PD1, CD2, CD3, CD5, CD7, CD8, CD4 (weakly positive), CD8 > CD4, TIA1 and Ki-67 40%^+^, and immunonegative for GrB, CD10, CD20, CD56 and CXCL-13 (Figure 1). Hodgkin’s lymphoma (HL) and T-cell non-Hodgkin’s lymphoma (NHL) were suspected. Consultation with Professor Xiaoge Zhou (Beijing China–Japan Friendship Hospital) revealed a diagnosis of CL (HL and cytotoxic T-cell lymphoma) complicated by Epstein–Barr virus (EBV) infection. The patient was transferred to the Department of Hematology at Shanxi Tumor Hospital. A bone marrow (BM) smear revealed a lymphoblastic cell proportion of 3.5%, and a bone marrow biopsy (BMB) showed T-cell involvement. The BM cells were immunopositive for CD3 and CD5, and immunonegative for CD10, CD30, CD56, PAX-5 and MUM-1. The results of flow cytometry (FCM) revealed the proportions of lymphocytic and CD3^+^ cells to be 36.4% and 30.3%, respectively, and BM cells expressed CD2, CD3, CD4^dim^, CD5, CD7, CD8 and CD4/CD8 (3.9%/61.8% = 0.06) (Figure 2). T-cell lymphoproliferative disorder was suspected. The EBV-DNA copy number was 3.84 × 10^3^ IU/mL. The patient was negative for cytomegalovirus (CMV) infection by PCR. The findings of chromosome 46XY and erythrocyte sedimentation (ESR) tests were normal. Immune function was low as determined by a natural killer (NK) proportion of 5.6%, a CD45^+^CD3^+^ proportion of 92.9%, a CD3^+^CD4^+^ proportion of 23.6%, a CD3^−^CD19^+^ proportion of 1.1% and an SIL-2R level of 493 U/mL. The clinical diagnosis was stage IVA CL, with an International Prognosis Index (IPI) score of 2 and an Eastern Cooperative Oncology Group (ECOG) score of 2, complicated by EBV infection. The patient received one cycle of adriamycin, bleomycin, vincristine (VCR) and dacarbazine (ABVD regimen) combined with chidamide, followed by one cycle of gemcitabine, cisplatin and dexamethasone (DEX) (GDP regimen) combined with chidamide and then oral acyclovir. The results of a PET-CT scan showed that the dimensions of the enlarged lymph node decreased partially (<50%). The EBV-DNA copy number was 5 × 10^2^ IU/mL. The patient achieved stable disease, but was lost to follow-up.

### 1.2 Case 2

**Figure 3:**
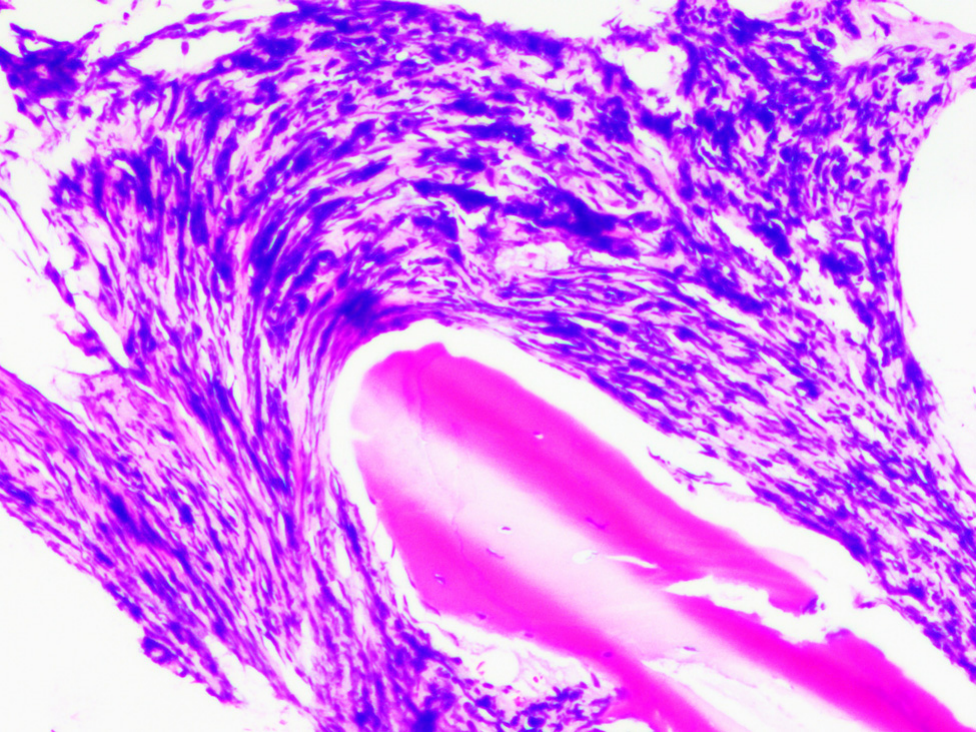
Histopathological findings of the BMB performed on case 2 showing moderate infiltration of cells between trabeculae (hematoxylin and eosin, 100×).

A 65-year-old female was admitted to the Department of Hematology at Shanxi Tumor Hospital in December 2015 with a 1-year history of bilateral cervical lymph node enlargement. She received penicillin for 10 days, but failed to respond to the antibiotic. The patient lost 10 kg of body weight. A cervical lymph node biopsy was performed at a local hospital in August 2015. Histopathology revealed lymphoid cell infiltration. Consultation with Professor Xiaoge Zhou revealed the infiltration of two types of lymphoid cells. The first population of medium-to-large cells had small nucleoli. The cells were immunopositive for CD20, PAX-5 and MUM-1. The second population of small cells had irregular nuclei and little cytoplasm. The cells were immunopositive for CD3, CD21, CXCL-13, PD-1 and Ki-67 30–40%^+^. PCR results revealed immunoglobulin heavy chain (Ig_HC_) and T-cell receptor delta (TCR-D) expression. The diagnosis was CL (diffuse large B-cell lymphoma [DLBCL] and angioimmunoblastic T-cell lymphoma [AITL]). The findings of the BM smear were normal, and those of the BMB showed BM involvement. Moderate infiltration of large heteromorphic cells was observed between trabeculae (Figure 3). These cells were immunopositive for CD3, CD20 and Ki-67 30%^+^, and the results of FCM revealed no abnormal expression in any biomarker. Laboratory findings showed an HGB level of 103 g/L, a WBC count of 9.6 × 10^9^/L and a PLT count of 267 × 10^9^/L. The levels of LDH, β2-MG and ES were 268 U/L, 3.29 mg/L and 40 mm/h, respectively. The results of liver and kidney functions, EBV and CMV infections, and coagulation tests were normal. A contrast-enhanced CT scan showed bilateral cervical, axillary, inguinal, mediastinal, retroperitoneal and para-iliac vessels, as well as lymphadenopathy and splenomegaly. The clinical diagnosis was stage IVsB CL, with an IPI score of 4 and an ECOG score of 2. The patient received eight cycles of cyclophosphamide, pirarubicin, VCR and DEX (CTOP regimen) combined with chidamide. The results of the PET-CT scan and BMB showed no abnormal findings, and the patient achieved complete remission. Nine months later, a CT scan showed enlargement of the left axillary lymph nodes and multiple nodules in the bilateral parotid glands. Relapse was considered. The patient refused a biopsy of the axillary lymph nodes. She received chidamide monotherapy for 3 months, which was then terminated. One year later, she underwent progressive disease (PD). A CT scan showed a mediastinal mass and bilateral pleural effusions. The patient succumbed to lymphoma 45 months after diagnosis.

## 2 Discussion

Composite lymphoma (CL) is a rare disease composed of two or more morphologically and immunophenotypically distinct lymphomas in the same organ or tissue. Its estimated incidence is 1–4.7% of all newly diagnosed lymphomas per year. Raufi et al. presented a CL patient who underwent biopsy of the supraclavicular lymph node, and the diagnosis was peripheral T-cell lymphoma (PTCL) and lymphoplasmacytic lymphoma [1]. Our two cases underwent biopsies of cervical lymph nodes, and the diagnosis was CL. Written informed consent was obtained from both patients. The study protocol was approved by the ethics committee of Shanxi Tumor Hospital, Shanxi, China. Case 1 was diagnosed with HL and cytotoxic T-cell lymphoma, whereas case 2 was diagnosed with DLBCL and AITL. However, the definition of CL has recently been expanded to include two distinct lymphomas presenting sequentially or simultaneously in different organs or tissues [1,2]. Guan et al. reported such a case of breast DLBCL and BMB, complicated by T-cell lymphoblastic lymphoma. For this case, PCR results revealed expression of TCR-G and Ig_HC_ [2].

Many types of tumors have been reported in CL, including multiple B-cell lymphomas, B-cell and T-cell lymphomas, HL and NHL. For example, Demurtas et al. presented seven CL cases of B-cell NHL + T-cell NHL, five CL cases of B-cell NHL + B-cell NHL, one CL case of T-cell NHL + T-cell NHL and four CL cases of NHL + HL [3]. Suefuji et al. reported 21 CL cases of DLBCL + AITL and 8 CL cases of T cell NHL + B-cell NHL [4], while Wang et al. reported 5 CL cases of PTCL + DLBCL [5]. However, we are the first to report a case of HL + cytotoxic T-cell lymphoma.

There are several possible mechanisms for the simultaneous occurrence of CL. One possibility is immune deficiency, as many cases complicated by EBV infection show immune dysfunction. Suefuji et al. presented 18 (67%) CL cases who were infected with the EBV [4], whereas Wang et al. reported 4 out of 9 (44%) patients who were infected [5]. Papalas et al. described a complicated case of primary cutaneous composite EBV-associated DLBCL and PTCL [6]. Case 1 was infected with the EBV, which showed involvement of R–S cells and cytotoxic T-cells, and his immune function was low. Furthermore, Sugimoto et al. reported a case of EBV-positive aggressive natural killer leukemia (ANKL), which was diagnosed on the basis of peripheral blood and BM examinations. However, an inguinal lymph node biopsy revealed EBV-positive cytotoxic T-cell lymphoma and the presence of a small number of EBV-positive ANKL cells. A diagnosis of EBV-positive CL was made. However, the disease was complicated by hemophagocytic syndrome, which has a very poor prognosis with a median survival time of less than 2 months. The patient died 8 days after admission. Nevertheless, there are no reports of EBV infection with involvement of NK cells or T-cells [7], and some CL cases are negative for EBV infection. The lack of EBV infections in some patients suggests that other mechanisms may promote the development of CL. External factors, including chronic viral infections, rheumatoid arthritis and celiac disease, are thought to predispose patients to the development of CL [4].

Due to the complexity of CL, most cases follow an aggressive clinical course. CL presents a unique challenge to clinicians as there are little data on treatment and outcomes. For example, Suefuji et al. reported 29 cases with CL. Three patients were not treated with chemotherapy due to their poor overall health, while 19 cases were treated with CHOP and R-CHOP regimens. Eleven (58%) patients achieved complete remission, 12 (63%) patients were alive and 7 (36%) patients died of the disease [4]. González-Gascón et al. presented two CL cases with mantle cell lymphoma (MCL) and PTCL that were treated with R-CHOP and R-Hyper CVAD regimens. Both patients achieved complete remission, but subsequently underwent autologous stem cell transplantation (ASCT). Case 1 remained in complete remission for 5 years, but eventually suffered a relapse with pancytopenia. The relapse was limited to the BM, and the results of laboratory tests suggested a late relapse of the MCL. The patient started treatment with ibrutinib and achieved a partial remission. Case 2 has remained disease-free for 4 years. Consolidation treatment with ASCT was performed after achieving first CR, which has achieved a long-term remission [8].

As such, effective treatment of CL requires consideration of both disease components. Chidamide, a selective inhibitor of histone deacetylase, is the first drug approved for the treatment of relapsed or refractory PTCL with good outcomes. Shi et al. presented 383 relapsed or refractory PTCL patients. For patients receiving chidamide monotherapy (*n* = 256), the disease control rate (DCR) was 64.45%. For patients receiving chidamide combined with chemotherapy (*n* = 127), the DCR was 74.02%. This large study demonstrates that chidamide has acceptable efficacy and safety profiles for relapsed and refractory PTCL patients [9]. In addition, Jin et al. described a refractory AITL case who received chidamide with chemotherapy and achieved complete remission [10]. Chidamide combined with chemotherapy may be a new treatment for refractory and relapsed PTCL and ALTL patients.
